# Advances and New Perspectives in Plant-Microbe Interactions

**DOI:** 10.3390/ijms24065143

**Published:** 2023-03-07

**Authors:** Marouane Baslam

**Affiliations:** 1 Laboratory of Biochemistry, Department of Applied Biological Chemistry, Faculty of Agriculture, University of Niigata, Niigata 950-2181, Japan; mmm.baslam@gmail.com; 2 Centre d’Agrobiotechnologie et Bioingénierie, Unité de Recherche Labellisée CNRST (Centre AgroBio-Tech-URL-CNRST-05), Université Cadi Ayyad, Marrakech 40000, Morocco; 3 Laboratory of Agro-Food, Biotechnologies and Valorization of Plant Bioresources (AGROBIOVAL), Department of Biology, Faculty of Science Semlalia, Cadi Ayyad University, Marrakesh 40000, Morocco

Plants, due to their sessile nature, are constantly exposed to a myriad of microorganisms. Plant–microbe encounters can be friendly and beneficial or hostile and harmful, depending on the nature of the interaction. Direct cooperative (symbiotic) plant–microbe relationships are dominant in many ecosystems. Synergistic interactions could determine crop health in the natural agroecosystem by providing numerous services to plants. By contrast, plants are also constantly exposed to fungal, bacterial, and/or viral pathogens, causing substantial economic losses, thereby leading to critical global food security pressure. As a result, in both relationships, a complex network of interactions has evolved within the plant–microbes, and unique defense mechanisms to fight infections, mediated by a multitude of chemical signals derived from both plants and microbes. Understanding and deciphering the molecular work to reveal the principles/fundamental processes that orchestrate plant–microbe interactions are the key goals of plant and microbe research. Additionally, plant–microbe interactions are profoundly affected by external environmental conditions. Understanding this “triangle” and how environmental conditions modulate plant–microbe interactions is crucial to predict the performance of plant–microbe interactions, engineer effective biofertilizers and/or biocontrol agents, and design “dream” crop plants with increased resilience or synthetic microbe communities for reproducible beneficial outputs, in order to address today’s challenges in the realm of human population growth, globalization, and current and future climate change.

This Special Issue spans a range of topics at different levels of organizations and key open questions in plant microbiome research to unravel the complex network of genetic, biochemical, physical, and metabolic interactions among the plant, the associated microbial communities, and the environment, as posed by 13 research groups with expertise ranging from eco-physiology to cell and molecular biology. A set of two in-depth reviews address our understanding of plant-associated microbiomes and their complex network of genetic, biochemical, physical, and metabolic interactions. Nadarajah and Abdul Rahman (2021) [1] highlight the aboveground and belowground microbial interactions, the development of diseases and emerging threats, the beneficial uses of microbes, and the new methods employed to determine the soil microbial structure, density, and function. These tools can structurally and functionally characterize more chemical signaling compounds and identify the role they play in plant–microbe interactions as well as microbe–microbe interactions. As a call to action for the plant microbiome research community, a comprehensive review by Abdul Hamid and Nadarajah (2022) [2] outlines the chemical signaling between bacteria–bacteria, bacteria–fungi, and plant–microbe and the diverse roles played by these compounds in plant–microbe interactions, and provides recommendations for researchers seeking to learn about the success of microbial chemicals in improving crop yield and growth. They also review the current and potential uses and significance of natural products in agriculture.

Two research articles in this issue address topics related to the composition, assembly, and dynamics of plant-associated microbial communities and the host functions they provide. Graziano et al. (2022) [3] show the effect of the combination of biochar (as a carrier), microbial consortia, and/or arbuscular mycorrhizal fungi (AMF) on soil microbial communities and the systemic changes induced in durum wheat and maize tissues. By merging a dataset of molecular and physiological data, the authors demonstrate the plant and soil crosstalk in reacting to different environmental conditions and plant age, and they produce a viable scenario for new plant fertilization. Illescas et al. (2022) [4] describe the use of a selected *Trichoderma* T140 (out of eight strains, representing different genotypes within the genus) in acclimatizing wheat plants under high N fertilization and water limitation conditions, and its role in maintaining grain yield and quality traits.

The field of plant–microbiome interaction under a changing world appears to have entered a golden age over the last few years, with multiple major discoveries being reported. Cheng et al. (2021) [5] provide insights into the molecular mechanism that allows *Epichloë gansuensis*, seed-borne endophytic fungi, to increase salinity tolerance in *Achnatherum inebrians* host grasses, by exploring the full-length transcriptome information. Understanding the interactions of native grasses, fungal endophytes and the transcriptional responses of the mutualistic symbiotic correlation will help to design breeding with endophytic fungi and develop a salt-tolerant forage in the future. Kataoka et al. (2022) [6] report on the isolation, identification, and characterization of endophytic bacteria in the halophyte common ice plants (*Mesembryanthemum crystallinum* L.). The inoculation of two multipotent isolates (*Streptomyces* spp. strain 2 and *Microbacterium* spp. strain 4) that produced plant growth-promoting (PGP) traits and salinity-ameliorating metabolites promoted the host plant growth and development under salinity stress conditions. These microorganisms are actively associated with varied cellular communication processes through quorum sensing and secondary metabolites, such as the production of Indole-3-acetic acid (IAA), exopolysaccharide (EPS) siderophore, ammonia, ACC deaminase, and the solubilization of phosphate. Bomle et al. (2021) [7] highlight the progress of the beneficial roles of PGP rhizobacteria with 1-Aminocyclopropane-1-Carboxylate (ACC) deaminase activity—which transforms ACC to ethylene—as green bio-inoculants in reducing the impact of saline conditions; this is particularly relevant as soil salinization affects a large percentage of croplands worldwide, and salt tolerance is an increasingly important breeding target. The authors also show the applications of ACC deaminase-producing PGPR as a beneficial tool in seed biopriming techniques.

An important class of abiotic interactions also covered in this issue is plant–microbe–heavy metals interactions, which are addressed in two papers. Raklami et al. (2022) [8] provide a deep dive into the progress and promising techniques for bioremediation and plant-associated microbe’s affair for environmental clean-up, address how the plant microbiome modulates heavy-metal(loid)s remediation and its molecular bases of working mechanisms, and provide insights into the key challenges and future directions for the remediation of metal(loid)s-polluted agricultural soils. Chlebek et al. (2022) [9] identify a new strain (*Pseudomonas qingdaonensis* ZCR6) isolated from the rhizosphere of maize, exhibiting the potential for the bioremediation of sites co-contaminated with hydrocarbons and heavy metals, biosurfactant production, and PGP.

Regarding pathogens and pests in plant biotic interactions, four of the papers in this issue focus on how plants recognize (shape the associated microbiomes) and defend against pathogens. Majumdar et al. (2022a) [10] uncover, for the first time, the sugar beet leaf bacteriome response against beet curly top virus (BCTV). Using BCTV-susceptible and—resistant sugar beet genotypes and beet leafhopper (BLH)—mediated natural BCTV infection, their study highlights the restructuring of the bacterial microbiome in the leaves following BCTV infection, and their differential regulation in both sugar beet genotypes in a temporal manner of infection stage. They identify potential bacterial biomarkers in the leaves of R genotypes that might play a potential role in resistance against the virus at the early infection stage. Taking a step further, Majumdar et al. (2022b) [11] provide a new molecular mechanism by which *Rhizoctonia solani*—which causes Rhizoctonia crown and root rot in sugar beet—interacts with *Leuconostoc mesenteroides* during sugar beet root infection and increases root damage. Through a combination of tools involving the use of purified enzymes, an analysis of cell-wall-degraded carbohydrates and their impact on total C and N in the cells, and a global mRNAseq analysis at early root infection stages, the authors revealed potential candidate genes and highly co-expressed gene clusters in the complex three-way interaction between sugar beet–*R. solani*–*L. mesenteroides*, that could potentially be critical in host defense and pathogenesis during the early root infection stages under field conditions. Tundo et al. (2022) [12] report an analysis of how *Fusarium graminearum* FGSG_03624 xylanase modulates plant immunity by acting as a PAMP, elicits defense responses, and increases pathogen resistance in plants. Yang et al. (2022) [13] show how chirality affects the activity and mechanism of action of prothioconazole (Pro), as a typical triazole fungicide; enantiomers on *Fusarium oxysporum* f. sp. cubense tropical race (TR4), the notorious virulent strain causing *Fusarium* wilt of banana; and the need to take the enantioselectivity into account for comprehensive risk assessments during fungal treatment for plant disease control. Their study revealed an enantioselective mechanism of Pro against TR4, which may rely on the enantioselective damages to the fungal cell membrane and the enantiospecific of cytochrome P450 14α-sterol demethylases binding affinity. Both articles provide the chemical basis for the rational design and development of peptides approach and novel biopesticides as strategies for pest control and crop protection.

This Special Issue presents examples of cross-disciplinary research that borrow concepts from multi-‘omics’, engineering, experimental biology, computational biology/statistics, and eco-physiology to illustrate how plant–microbiome research can help to mitigate (a)biotic stresses and enhance food security. We hope these articles serve to inform and inspire researchers in key areas of plant microbial research. We encourage authors to continue to submit their best work in molecular plant sciences to *The International Journal of Molecular Sciences*. Articles in this area will be added to an online collection of articles on molecular plant sciences and plant–microbe interactions, building on the articles presented in this Special Issue.
